# Durability of serologic responses to inactivated hepatitis A virus vaccination among people living with HIV following acute hepatitis A outbreak: a 5-year follow-up study

**DOI:** 10.1080/22221751.2023.2239946

**Published:** 2023-07-31

**Authors:** Kuan-Yin Lin, Hsin-Yun Sun, Yu-Shan Huang, Wang-Da Liu, Szu-Min Hsieh, Sung-Hsi Huang, Guan-Jhou Chen, Chien-Ching Hung

**Affiliations:** a Department of Internal Medicine, National Taiwan University Hospital and National Taiwan University College of Medicine, Taipei, Taiwan; b Center of Infection Control, National Taiwan University Hospital, Taipei, Taiwan; c Department of Medicine, National Taiwan University Cancer Center, Taipei, Taiwan; d Department of Internal Medicine, National Taiwan University Hospital Hsin-Chu Branch, Hsinchu, Taiwan; e Department of Tropical Medicine and Parasitology, National Taiwan University College of Medicine, Taipei, Taiwan; f Min-Sheng General Hospital, Taoyuan, Taiwan; g Department of Internal Medicine, National Taiwan University Hospital Yunlin Branch, Yunlin, Taiwan

**Keywords:** Viral hepatitis, immunogenicity, seroprotection, seroreversion, men who have sex with men

## Abstract

Serologic responses to hepatitis A virus (HAV) vaccination may wane among immunocompromised populations. To evaluate the long-term seroresponses to 2-dose HAV vaccination, we retrospectively included people living with HIV (PLWH) who had achieved seroconversion within 12 months after vaccination at a university hospital during an outbreak of acute hepatitis A between 2015 and 2017. PLWH included in the study received either Havrix or Vaqta. The seroresponses were evaluated 60 months after the second dose of vaccination and estimated by the intention-to-treat (ITT) with last-observation-carried-forward (LOCF) and per-protocol (PP) analyses. Overall, 986 PLWH (median age, 34 years and CD4 count, 587 cells/µL) were included. The rates of PLWH with persistent seroprotection at month 60 of vaccination were 90.7% (894/986) and 97.4% (748/768) in the ITT with LOCF and PP analyses, respectively. PLWH with persistent seroprotection had achieved higher peak anti-HAV IgG titers after vaccination and had a slower decline in antibody levels compared with those with seroreversion. In the multivariable analysis, seroreversion at month 60 was associated with a higher body-mass index (per 1-kg/m^2^ increase, AOR, 1.10; 95% CI, 1.04-1.17), lowest-ever CD4 count (per 10-cell/µL increase, AOR 0.98; 95% CI, 0.97-1.00), plasma HIV RNA <200 copies/ml at vaccination (AOR, 0.28; 95% CI, 0.14-0.59), and having received Vaqta as the first dose of HAV vaccination (AOR, 0.44; 95% CI, 0.27-0.70). The seroprotection against HAV remained high in the long-term follow-up among PLWH on antiretroviral therapy after 2-dose HAV vaccination. Regular monitoring of seroresponses and timely administration of HAV vaccines are warranted to maintain seroprotection.

## Introduction

Hepatitis A virus (HAV) is transmitted primarily through ingestion of contaminated food and water, but was recently considered as one of the aetiologies for non-classical sexually transmitted infections [1]. Large outbreaks of acute hepatitis A (AHA) disproportionately impacting men who have sex with men (MSM) occurred worldwide during 2015-2020, especially in Taiwan, European countries, and the United States [2]. The worldwide distribution of AHA outbreaks may be linked to transmission through both sexual networks and international travel [3,4]. The widespread outbreaks had resulted in spillover to the general population, and people using drugs and being homeless were most heavily affected populations in the United States [5,6]. With shared transmission routes, people living with HIV (PLWH) are also at increased risk for HAV infection.

The most effective strategy for preventing HAV infection is HAV vaccination. In the general population, at least one dose of HAV vaccination could achieve a seroconversion rate of >95% and reduce >90% of incident AHA cases in endemic areas [7,8]. A mathematical modelling suggested that immune response to inactivated HAV vaccine might persist in ≥95% and ≥90% of the vaccinees for 30 and 40 years, respectively [9]. During the AHA outbreaks predominantly occurring among PLWH, HAV vaccination remained highly effective at both individual and population levels (96% and 81%, respectively) [10,11]. Nevertheless, serologic responses and durability of HAV vaccination are reduced among PLWH [12]. The delayed and suboptimal serologic responses to HAV vaccination were observed before the second dose of HAV vaccination among PLWH, with only around 60% mounting serologic responses at 6 months of vaccination [10]. Among the primary responders, 3.9% lost their seroprotection within 1.7 years and 75-88% could maintain long-term seroprotection at 5 years of vaccination [12,13]. Due to a poorer durability, booster HAV is recommended every 10 years for PLWH at continued risks of exposure [14]. A single-dose HAV revaccination among PLWH with waning immune responses after primary vaccination has been demonstrated to elicit rapid and sufficient seroresponses [15,16].

Previous studies have identified factors associated with immunogenicity and durability of HAV vaccination in at-risk populations; younger age, a weight lower than 70 kg, and improved immune status (e.g. a CD4 count >350 cells/µL and plasma HIV RNA load [PVL] < 200 copies/mL) have been shown to be related to better responses to HAV vaccination [12,13]. Different approaches have been adopted to enhance immunogenicity and durability of serologic responses to HAV vaccination among PLWH, including increased number of vaccine doses and using different vaccines. For example, the geometric mean concentrations (GMCs) of anti-HAV immunoglobulin G (IgG) at short-term and long-term follow-up were significantly higher for those receiving 3 doses of HAV vaccine than those receiving 2 doses [17,18]. While different inactivated HAV vaccines, such as Havrix and Vaqta, achieved comparable seroconversion rates among healthy individuals in randomized controlled trials [19,20], our previous study of different combinations of HAV vaccines demonstrated that Vaqta elicited faster and better serologic responses than that of Havrix before the second dose of HAV vaccine was administered among PLWH [21]. The effect of different HAV vaccine combinations on the durability of seroresponses remains unclear. In the present study, we aimed to evaluate the long-term seroresponses to inactivated HAV vaccination among PLWH following AHA outbreak.

## Methods

### Study setting and population

During 2015 and 2017, an HAV vaccination campaign was implemented to curb the unprecedented AHA outbreak predominantly affecting MSM living with HIV in Taiwan [11,22]. All PLWH seeking care at the National Taiwan University Hospital were screened for anti-HAV IgG, and those testing negative for anti-HAV IgG were advised to receive 2 doses of HAV vaccine administered 6 months apart. The vaccine shortages during the outbreak prompted the substitution of 1440 enzyme-linked immunosorbent assay units of Havrix (GlaxoSmithKline, Biologicals, Rixensart, Belgium) with 50 units of Vaqta (Merck, West Point, PA) [10,21]. Therefore, PLWH included in the study received either Havrix for both doses, Havrix as the first dose followed by Vaqta as the second dose, or Vaqta for both doses.

This retrospective study was conducted at the National Taiwan University Hospital to include adult PLWH who had achieved seroconversion within 12 months after the second dose of HAV vaccination between 1 June, 2015 and 31 December, 2017. PLWH who had had a history of HAV infection or no follow-up measurements of anti-HAV IgG beyond 12 months after the second dose of HAV vaccination were excluded. In accordance with the national HIV treatment guidelines, PLWH returned for assessment of virological, immunological, and clinical status every 3–6 months. Serologies of viral hepatitis, including HAV, hepatitis B virus (HBV), hepatitis C virus (HCV), were tested every 6–12 months [23]. This study was approved by the Research Ethics Committee of the hospital (201605103RINC), and informed consent to collection of clinical data was waived.

### Outcomes and follow-up

The anti-HAV IgG antibody persistence was evaluated 5 years after HAV vaccination. The primary outcome was serologic response at month 60 of the second dose of HAV vaccination, and the secondary outcomes were the serologic responses between 12 and 48 months after the second HAV vaccination. All participants were followed until occurrence of HAV seroreversion (loss of serologic response), AHA, loss to follow-up, or the end of this study on 31 December, 2022, whichever occurred first. Participants with seroreversion were suggested to receive at least a single-dose of HAV revaccination.

### Laboratory investigations

Serum anti-HAV IgG levels were assessed using a semi-quantitative immunoassay (ARCHITECT HAVAb-IgG; Abbott Diagnostics, Wiesbaden, Germany) that measured the chemiluminescent reaction. The chemiluminescent signal was measured in signal-to-cutoff (S/CO) values by comparing relative light units (RLU) between the test sample and a cutoff determined from an ARCHITECT calibration. A specimen with a S/CO value ≥1.00 was regarded as being positive for anti-HAV IgG, and there was a direct correlation between serum anti-HAV IgG titers and RLUs [24]. The diagnosis of AHA was based on clinical manifestations and the presence of positive anti-HAV IgM detected with the use of chemiluminescence immunoassay (ARCHITECT HAVAb-IgM; Abbott Diagnostics). Data on the lowest-ever CD4 counts before HAV vaccination, antiretroviral therapy, and follow-up CD4 and PVLs were collected at and after HAV vaccination. HBV surface antigen and anti-HCV antibody were determined using an enzyme immunoassay (Abbott Laboratories, Abbott Park, IL). As part of routine care for PLWH, regular serological evaluations for syphilis were conducted every 3–6 months; incident syphilis was used as a surrogate marker for at-risk sexual behaviour. Syphilis was diagnosed based on reactive serologic tests, which included the rapid plasma reagin test (BD Macro-VueTMRPR Card tests) and the *Treponema pallidum particle* agglutination test (FTI-SERODIA-TPPA; Fujirebio Taiwan Inc., Taoyuan, Taiwan).

### Statistical analysis

Categorical variables were compared using either Fisher’s exact test or the chi-squared test, while the Wilcoxon–Mann–Whitney test was used to analyze continuous variables. We estimated the serologic responses using both intention-to-treat (ITT) and per-protocol (PP) analyses. In the ITT analysis, missing data on anti-HAV IgG at month 60 were imputed using the last-observation-carried-forward (LOCF) approach. To identify factors associated with seroreversion at month 60 of the second dose of HAV vaccination, logistic regression analysis was conducted with time-updated covariates such as CD4 count and PVL. Variables with a *P* value <0.1 were included in the multivariable model with backward selection to obtain the adjusted odds ratio (AOR) and corresponding 95% confidence intervals (CIs) for each variable. The Kaplan-Meier method and log-rank test were used to compare the cumulative proportions of PLWH who experienced seroreversion following HAV vaccination. Cox regression analysis with time-updated covariates was used to identify predictors of incident seroreversion and to estimate the adjusted hazard ratio (AHR) for each variable. Variables with a *p*-value <0.05 were considered statistically significant. All statistical analyses were performed using STATA software version 17.0 (Stata Corporation, College Station, TX).

## Results

**Figure 1.**
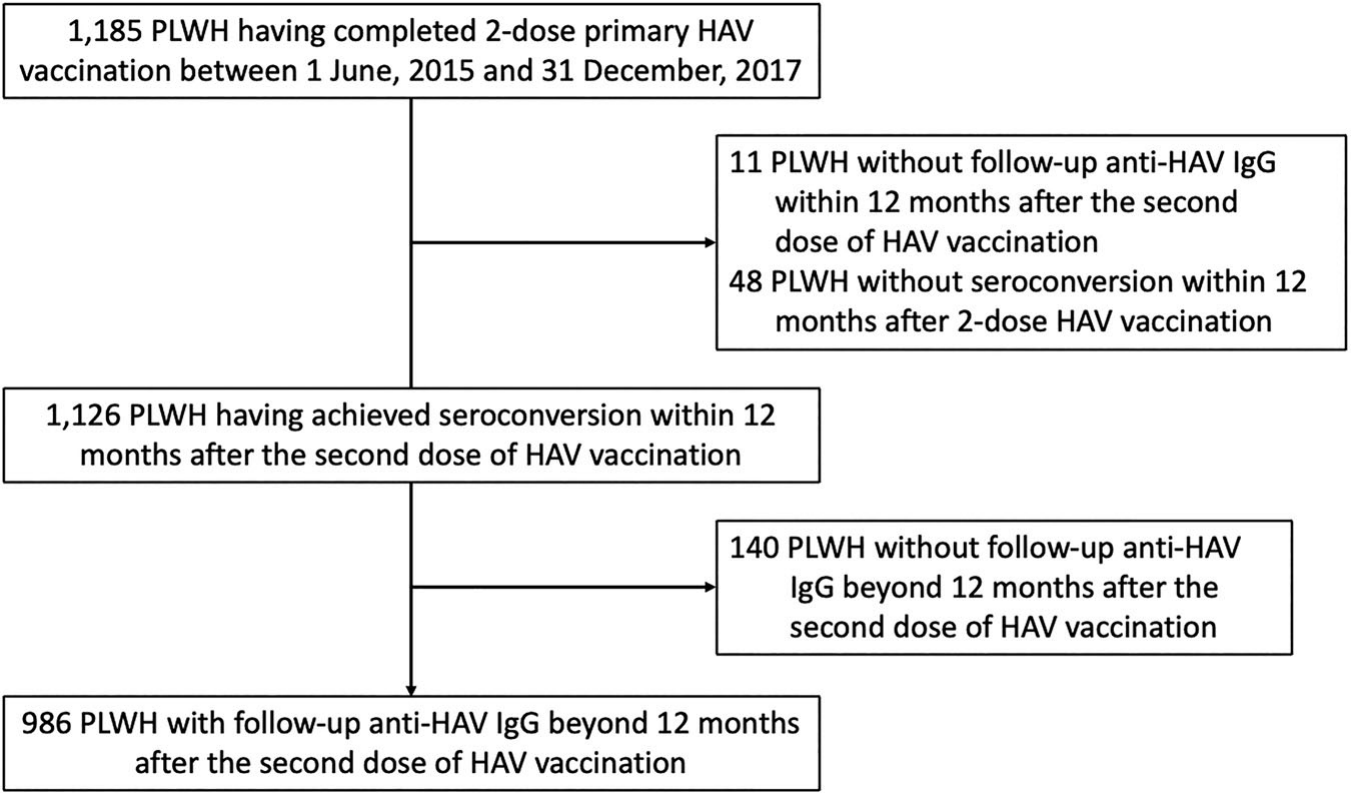
Study flow. Abbreviations: HAV, hepatitis A virus; IgG, immunoglobulin G; PLWH, people living with HIV.

From 1 June 2015–31 December 2017, 1,185 PLWH had completed 2-dose primary HAV vaccination and 1,126 (95.0%) PLWH had achieved seroconversion within 12 months after the second dose of HAV vaccination; and 986 (87.6%) with follow-up anti-HAV IgG beyond 12 months after the second dose of HAV vaccination were included (Figure 1). The majority of included PLWH were MSM (95.5%) with a median age of 34 years (interquartile range [IQR], 29–40) and body-mass index (BMI) of 22.6 kg/m^2^ (IQR, 20.7–24.7). At the time of HAV vaccination, more than 95% of PLWH were receiving combination antiretroviral therapy with a median baseline CD4 count of 587 cells/µL and 92.4% having achieved PVL to <200 copies/mL (Table 1). At the end of follow-up, the median CD4 count had increased to 667 cells/µL and the proportion of PLWH with PVL <200 copies/mL to 98.2%.

**Table 1.** Clinical characteristics of included PLWH with and those without seroreversion at month 60 of the second dose of HAV vaccination.

	Overall (*n* = 986)	With seroreversion (*n* = 92)	Without seroreversion (*n* = 894)	*P*
Age, median (IQR), years	34 (29-40)	36 (32-43)	34 (28-40)	0.001
Male sex, *n* (%)	973 (98.7)	91 (98.9)	882 (98.7)	0.838
Men who have sex with men, n (%)	942 (95.5)	84 (91.3)	858 (96.0)	0.039
Weight, median (IQR), kg	67 (60-75)	71 (63-77)	66 (60-74)	0.001
Body-mass index, median (IQR), kg/m^2^	22.6 (20.7-24.7)	23.8 (21.4-26.1)	22.5 (20.7-24.6)	0.002
HBsAg positivity, *n* (%)	88 (8.9)	13 (14.1)	75 (8.4)	0.066
Anti-HCV positivity, *n* (%)	61 (6.2)	10 (10.9)	51 (5.7)	0.050
Receiving immunosuppressant^a^, *n* (%)	3 (0.3)	1 (1.1)	2 (0.2)	0.152
Receiving ART at vaccination, *n* (%)	945 (95.8)	89 (96.7)	856 (95.7)	0.651
Lowest-ever CD4 count, median (IQR), cells/µL	266 (133-386)	172 (70-330)	273 (150-387)	0.003
CD4 count at vaccination, median (IQR), cells/µL	587 (452-764)	510 (407-679)	592 (458-766)	0.003
PVL at vaccination, median (range), log copies/mL	UD^b^ (UD-6.3)	UD (UD-5.4)	UD (UD-6.3)	0.011
PVL <200 copies/ml at vaccination, *n* (%)	909 (92.4)	80 (87.0)	829 (92.7)	0.040
CD4 count at the end of follow-up, median (IQR), cells/µL	667 (519-855)	557 (454-741)	676 (529-866)	0.001
PVL at the end of follow-up, median (range), log copies/mL	UD (UD-5.6)	UD (UD-4.4)	UD (UD-5.6)	0.452
PVL <200 copies/mL at the end of follow-up, *n* (%)	968 (98.2)	89 (96.7)	879 (98.3)	0.280
Combinations of HAV vaccine, *n* (%)				0.009
Havrix-Havrix	19 (1.9)	2 (2.2)	17 (1.9)	
Havrix-Vaqta	351 (35.6)	46 (50.0)	305 (34.1)	
Vaqta-Vaqta	616 (62.5)	44 (47.8)	572 (64.0)	
Peak anti-HAV IgG titers, median (IQR), S/CO	10.1 (7.6-11.8)	4.7 (2.8-6.4)	10.4 (8.7-12.1)	<0.001
Syphilis during follow-up, *n* (%)	348 (35.3)	39 (42.4)	309 (34.6)	0.135

^a^ Including concurrent use of chemotherapy and immunomodulation agents.

^b^ UD, < 20 copies/mL.

Abbreviations: ART, antiretroviral therapy; HAV, hepatitis A virus; HBsAg, hepatitis B surface antigen; HCV, hepatitis C virus; IgG, immunoglobulin G; IQR, interquartile range; PLWH, people living with HIV; PVL, plasma HIV RNA load; UD, undetectable; S/CO, signal-to-cutoff.

Due to an early shortage of Havrix, only 19 (1.9%) of the included PLWH received Havrix for both doses (Havrix-Havrix), 351 (35.6%) received Havrix as the first dose followed by Vaqta as the second dose (Havrix-Vaqta), and 616 (62.5%) received Vaqta for both doses (Vaqta-Vaqta). The overall peak anti-HAV IgG titers within 12 months after 2-dose HAV vaccination were 10.1 S/CO (IQR, 7.6–11.8), which were highest in PLWH receiving Vaqta-Vaqta (10.6 S/CO), followed by those receiving Havrix-Vaqta (9.3 S/CO) and those receiving Havrix-Havrix (8.6 S/CO). A high rate of incident syphilis (35.3%) was observed during the 5-year follow-up.

The clinical characteristics of 140 PLWH who were not included in this study due to the lack of follow-up anti-HAV IgG beyond 12 months after 2-dose HAV vaccination were generally similar to those who were included, except that the included PLWH were younger (median age, 34 vs 36 years), more likely to have received Havrix-Vaqta (35.6% vs 15.7%), and mounted lower peak anti-HAV IgG titers (median, 10.1 vs 10.6 S/CO) (Supplementary Table S1).

The percentage of persistent responders at month 60 of 2-dose of HAV vaccination were 90.7% (894/986) in the ITT analysis using the LOCF approach and 97.4% (748/768) in the PP analysis. Compared with 894 PLWH with persistent seroresponses at months 60, 92 seroreverters were older (median age, 36 vs 34 years) and less likely to be MSM (91.3% vs 96.0%), had a higher BMI (median, 23.8 vs 22.5 kg/m^2^), lower CD4 counts (lowest-ever, 172 vs 273 cells/µL; at vaccination, 510 vs 592 cells/µL; and at the end of follow-up, 557 vs 675 cells/µL), and a lower percentage of PVL <200 copies/mL at vaccination, were more likely to have received Havrix as the first dose followed by Vaqta as the second dose (50.0% vs 34.1%), and mounted lower peak anti-HAV IgG titers (median, 4.7 vs 10.4 S/CO) (Table 1). In multivariable analysis, seroreversion at month 60 of HAV vaccination was associated with a higher BMI (per 1-kg/m^2^ increase, AOR, 1.10; 95% CI, 1.04-1.17), lowest-ever CD4 count (per 10-cells/µL increase, AOR 0.98; 95% CI, 0.97-1.00), PVL <200 copies/ml at vaccination (AOR, 0.28; 95% CI, 0.14-0.59), and having received Vaqta as the first dose of HAV vaccine (AOR, 0.44; 95% CI, 0.27-0.70) (Table 2).

**Table 2.** Factors associated with seroreversion at month 60 of the second dose of HAV vaccination

	Univariable		Multivariable	
	OR (95% CI)	*P* value	AOR^a^ (95% CI)	*P* value
Age, per 1-year increase	1.04 (1.02–1.07)	0.002	1.00 (0.99–1.00)	0.975
Male sex	1.24 (0.16–9.63)	0.838		
Body-mass index, per 1-kg/m^2^ increase	1.10 (1.04–1.16)	0.002	1.10 (1.04–1.17)	0.002
HBsAg positivity	1.80 (0.95–3.38)	0.069	1.56 (0.78–3.13)	0.209
Anti-HCV positivity	2.02 (0.99–4.12)	0.055	1.74 (0.72–4.23)	0.217
Receiving immunosuppressant^b^	4.90 (0.44–54.58)	0.196		
Receiving ART at vaccination	1.32 (0.40–4.35)	0.652		
Lowest-ever CD4 count, per 10-cells/µL increase	0.98 (0.97–0.99)	0.004	0.98 (0.97–1.00)	0.044
CD4 count at vaccination, per 10-cells/µL increase	0.99 (0.98–1.00)	0.010	1.00 (0.99–1.01)	0.850
PVL <200 copies/ml at vaccination	0.51 (0.26–0.98)	0.042	0.28 (0.14–0.59)	0.001
CD4 count at the end of follow-up, per 10-cells/µL increase	0.99 (0.98–0.99)	0.003	0.99 (0.98–1.00)	0.120
PVL <200 copies/ml at the end of follow-up	0.51 (0.14–1.78)	0.289		
Combinations of HAV vaccine				
Havrix-Havrix	Reference			
Havrix-Vaqta	1.28 (0.29–5.73)	0.745		
Vaqta-Vaqta	0.65 (0.15–2.92)	0.578		
First dose of HAV vaccine, Vaqta vs. Havrix	0.52 (0.34–0.79)	0.003	0.44 (0.27–0.70)	0.001
Second dose of HAV vaccine, Vaqta vs. Havrix	0.87 (0.20–3.84)	0.857		
Syphilis during follow-up	1.39 (0.90–2.15)	0.136		

^a^ The ORs are the estimates of the effect of covariates on seroreversion at month 60 of vaccination, adjusted for age, body-mass index, HBsAg positivity, anti-HCV positivity, lowest-ever CD4 count, CD4 count and PVL at vaccination, CD4 count at the end of follow-up, and first dose of HAV vaccine using logistic regression model.

^b^ Including concurrent use of chemotherapy and immunomodulation agents.

Abbreviations: AOR, adjusted odds ratio; ART, combination antiretroviral therapy; HAV, hepatitis A virus; HBsAg, hepatitis B surface antigen; HCV, hepatitis C virus; OR, odds ratio; PVL, plasma HIV RNA load; S/CO, signal-to-cutoff.

**Figure 2.**
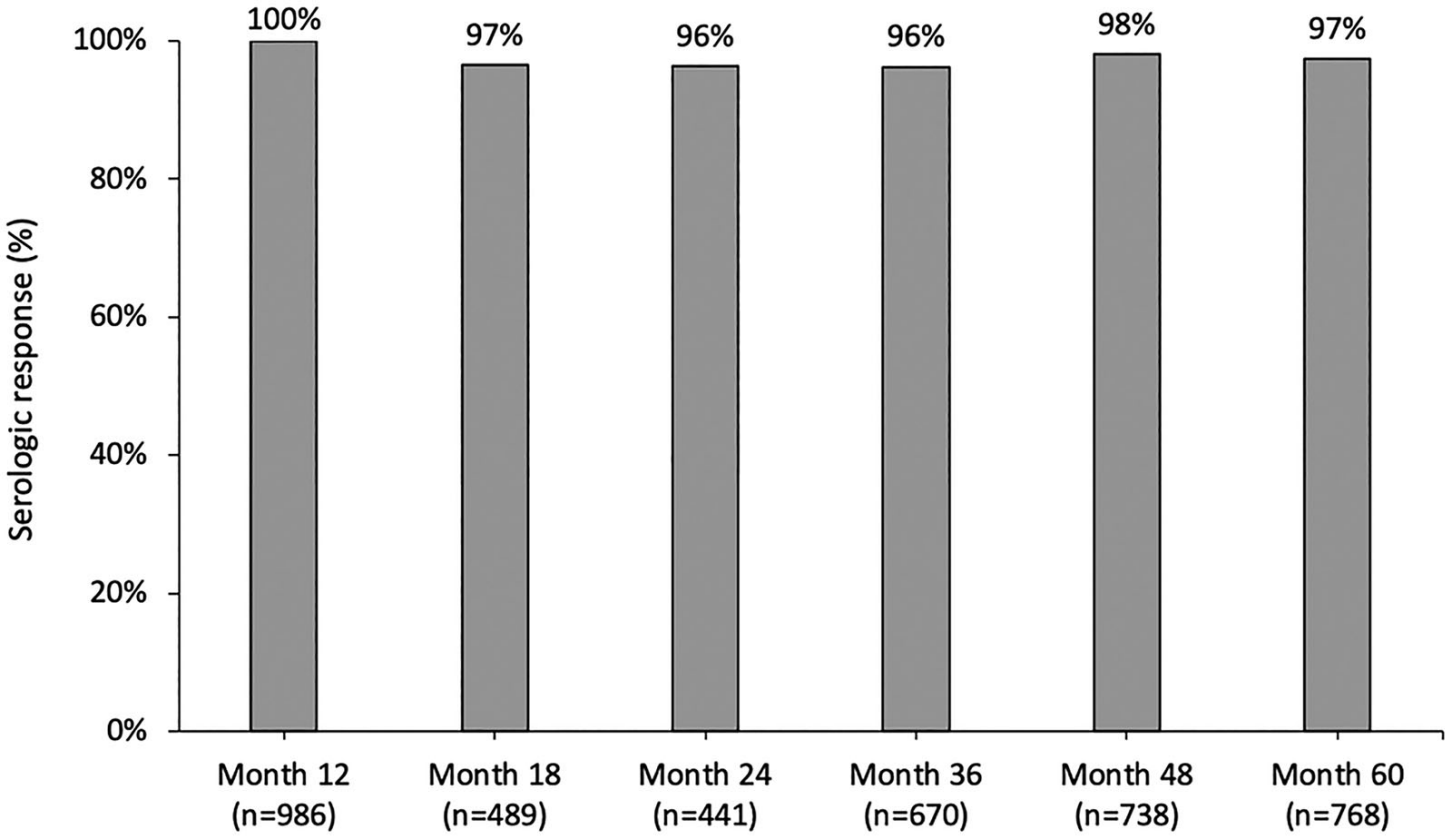
Percentages of persistent responders in 60 months of the second dose of HAV vaccination in the per-protocol analysis. *n, number of individuals with available test results of anti-HAV IgG. Abbreviations: HAV, hepatitis A virus; IgG, immunoglobulin G.

Although anti-HAV IgG measurements were taken following clinical care practices and not at fixed time points, the rates of PLWH with persistent seroprotection after vaccination at different follow-up intervals in the PP analysis consistently ranged from 96% to 98% between 12 and 48 months after 2-dose HAV vaccination (Figure 2). Evolution of anti-HAV IgG titers with time is illustrated in Supplementary Figure S1. While the seroreverters mounted lower anti-HAV IgG titers that waned early after HAV vaccination, the included PLWH with persistent seroprotection achieved higher anti-HAV IgG titers and had a gradual decline in antibody levels. None of the included PLWH acquired acute hepatitis A during the study period.

Time to seroreversion after the second dose of HAV vaccination was compared between PLWH having received different 2-dose combinations of HAV vaccines (Supplementary Figure S2). While the median time to seroreversion in PLWH having received Vaqta-Vaqta, Havrix-Vaqta, and Havrix-Havrix was 42.4, 37.2, and 24.9 months, respectively; the median time to seroreversion was statistically significantly longer in PLWH having received Vaqta as the first dose (42.5 months; IQR 31.2–57.9) than those having received Havrix as the first dose (35.8 months; IQR 20.2–48.2) (log-rank test, *P *= 0.002). In multivariable analysis, the factors associated with incident seroreversion within 60 months of the second dose of HAV vaccination included a higher BMI (per 1-kg/m^2^ increase, AHR, 1.10; 95% CI, 1.04-1.16), PVL <200 copies/ml at vaccination (AHR, 0.32; 95% CI, 0.17-0.60), and having received Vaqta as the first dose of HAV vaccine (AHR, 0.45; 95% CI, 0.29-0.71) (Supplementary Table S2). The findings were consistent with the estimates of the effect of covariates on seroreversion at month 60 of HAV vaccination in the logistic regression analysis.

## Discussion

In this 5-year follow-up study, we found that seroprotection against HAV infection remained >90% among PLWH who had undergone 2-dose HAV vaccination while on effective antiretroviral therapy. A lower BMI, HIV virologic suppression at vaccination, and having received the first dose of HAV vaccine with Vaqta were independently associated with long-term seroresponses against HAV infection. PLWH with persistent seroprotection mounted higher anti-HAV IgG titers and had a slower decline in the antibody levels than the seroreverters.

The rates of PLWH with persistent seroprotection were estimated 88.6%−100% after 1 year of vaccination, 86.8%−90% after 3 years, 85%−85.4% after 4 years, and 75.5%−88.4% after 5 years [12]. A prospective study conducted in Poland determined the long-term seroresponses after HAV vaccination among PLWH with 37% having been receiving ART and achieved a median CD4 count of 450 cells/µL. Among 49 PLWH who were HAV-seropositive at 1 year of vaccination and had available follow-up anti-HAV IgG, 75.5% remained seropositive at 5 years of HAV vaccination [25]. Another prospective study conducted in Taiwan revealed that, after 5 years of HAV vaccination, seroprotection persisted in 79% vs 76% and 94% vs 88% of the 3- vs 2-dose primary responders in the ITT and PP analyses, respectively [18]. The demonstration of waning immunity in a non-outbreak setting in these studies implies that regular monitoring of anti-HAV IgG and booster vaccinations for those at continued risk are warranted [14–16]. During the AHA outbreak among PLWH in Taiwan, our multicenter, retrospective study of 1,256 PLWH with seroresponses to primary vaccination also showed that 3.9% of primary responders seroreverted after a median follow-up of 1.7 years. The seroreversion rate during the outbreak was lower compared with that in a non-outbreak setting, which may be attributed to the natural booster effect during the outbreak and improved immune status at vaccination among the included PLWH [13]. In our study evaluating the durability of seroresponses to HAV vaccination during the AHA outbreak, the long-term seroreversion rates were also lower when compared to a setting without an outbreak.

The optimal timing of serologic response monitoring and booster vaccination has not yet been evaluated. Only the British HIV Association (BHIVA) recommends administering booster vaccination every 10 years, whereas other health authorities recommend periodic monitoring of anti-HAV IgG and administering booster vaccinations to individuals who remain at continued risk after seroconversion [12,14]. Regarding revaccination for seroreverters, a case–control study found a high seroresponse rate of 93.3% before administering the second dose of revaccination [15]. Furthermore, a recent randomized clinical trial demonstrated comparable seroresponse rates among PLWH with seroreversion who received either a one-dose or an accelerated two-dose schedule for HAV revaccination (97.7% vs 97.4% at week 24, respectively). These findings suggest that an additional dose of the HAV vaccine could effectively boost its immunogenicity among PLWH having experienced seroreversion [16].

The factors associated with persistent seroprotection observed in the previous studies included better immune status at vaccination, a lower weight, incident syphilis, absence of acute hepatitis C, and higher seroresponses to primary HAV vaccination [12]. The previous prospective study comparing seroresponses between 2-dose and 3-dose primary responders revealed that the administration of an additional dose of HAV vaccine led to an increase in GMCs of anti-HAV IgG, which persisted for a longer period before declining to subthreshold levels [18]. In the study assessing early seroreversion during the outbreak, PLWH with persistent seroprotection had faster serologic responses and higher peak anti-HAV IgG titers after HAV vaccination compared with those with seroreversion [13]. The shortages of vaccine supplies during the outbreak provided us with an opportunity to investigate the serologic responses to different vaccine combinations. While our previous study concluded the interchangeability of different HAV vaccine combinations since PLWH receiving Havrix-Vaqta and Vaqta-Vaqta combinations achieved similar seroresponse rates at week 48 of HAV vaccination, the Vaqta-Vaqta combination elicited significantly higher titers of anti-HAV IgG compared with the Havrix-Vaqta combination, suggesting Vaqta may be more immunogenic [21]. By extending the observation, this study also showed consistent findings that Vaqta might confer the long-term durability of seroprotection through achieving higher peak anti-HAV IgG titers in PLWH.

Apart from the inherent challenges of comparing the quantities of viral protein between Vaqta and Havrix, the immunoassays that were utilized to assess anti-HAV IgG titers in pivotal studies were different [7,8]. In the healthy population, a clinical trial was conducted to examine the boosting effect of Havrix and Vaqta in individuals who had previously received a single-dose of Havrix. The study revealed that Vaqta tended to result in numerically higher serologic responses (86% vs 80%) and anti-HAV IgG titers (3,274 vs 2,423 mIU/mL) [26]. In another randomized trial, seroconversion was achieved in participants receiving 3 different inactivated HAV vaccines, including Avaxim, Epaxal, and Havrix; however, the GMCs of anti-HAV IgG in the Havrix group were also significantly lower than that in the Avaxim group [27]. The factors that contribute to the production of anti-HAV antibodies after administration of different vaccines are still not fully understood. Since PLWH generally have weaker serologic responses to vaccination than the general population, the differences in serologic responses to different types of vaccines may be more prominent among PLWH and thus warrant further investigations.

Strengths of this study are a significant number of PLWH being followed up and high HAV vaccine coverage during the outbreak, which allows for better estimates of long-term seroresponses. However, the study has some limitations and caution in interpretation of the results is needed. First, selection bias is anticipated as only PLWH who had follow-up of serologic responses were included. Indeed, 12.4% (140/1,126) of PLWH who had seroconversion after completing 2 doses of HAV vaccination were not included for lacking anti-HAV IgG measured beyond 12 months after HAV vaccination. The excluded PLWH were more likely to have received Havrix-Vaqta combination with lower peak anti-HAV IgG titers, which could lead to a slight overestimation of long-term seroresponses. However, the majority of the clinical characteristics were similar between included PLWH and those who were not included. Second, the included PLWH were primarily males who were at-risk for AHA and had been receiving stable antiretroviral therapy. Hence cautions should be exercised to generalize our findings to women living with HIV, people using drugs and being homeless, and PLWH who have not initiated ART or have experienced virological failure [2]. Third, due to vaccine shortages, 2 types of HAV vaccines were administered and only 19 PLWH received 2 doses of Havrix, which precluded us from precisely estimating the durability of serologic response to this vaccine combination. Finally, the observed seroresponses could be attributed to either vaccination or natural infection, even though the outbreak was contained in 2017 and syphilis was used as a surrogate marker for at-risk sexual behaviour.

In conclusion, PLWH who were on stable antiretroviral therapy and had received 2-dose HAV vaccination during the outbreak could maintain a high level of long-term seroprotection against HAV, which might be further enhanced with administration of Vaqta. Regular monitoring of seroresponses and booster vaccination after primary HAV vaccination are still warranted, especially in PLWH with a higher BMI and virologic non-suppression.

## Supplementary Material

Supplemental Material
