# Supplementary material for: Durability of serologic responses to inactivated hepatitis A virus vaccination among people living with HIV following acute hepatitis A outbreak: a 5-year follow-up study
Source: Emerg Microbes Infect. 2023 Jul 31;12(2):2239946. doi: 10.1080/22221751.2023.2239946 (PMC10392330; doi:10.1080/22221751.2023.2239946)
Supplement: Supplemental Material [file TEMI_A_2239946_SM7411.docx]

**Supplementary Data**

Supplement to:

**Durability of serologic responses to inactivated hepatitis A virus vaccination among people living with HIV following acute hepatitis A outbreak: a 5-year follow-up study**

Kuan-Yin Lin, Hsin-Yun Sun, Yu-Shan Huang, Wang-Da Liu, Szu-Min Hsieh, Sung-Hsi Huang, Guan-Jhou Chen, Chien-Ching Hung

Contents:

Table S1. Clinical characteristics of included PLWH and those who were not included due to the lack of follow-up anti-HAV IgG beyond 12 months after the second dose of HAV vaccination

Table S2. Factors associated with incident seroreversion within 60 months of the

second dose of HAV vaccination

Figure S1. Evolution of serologic responses after the second dose of HAV vaccination

according to the status of seroreversion at month 60 of the second dose of HAV vaccination

Figure S2. Kaplan-Meier estimates of time to seroreversion after the second dose of

HAV vaccination. (A) Comparison between PLWH having received different 2-dose combinations of HAV vaccines. (B) Comparison between PLWH having received different first-dose HAV vaccines.

**Supplementary Table S1.** Clinical characteristics of included PLWH and those who were not included due to the lack of follow-up anti-HAV IgG beyond 12 months after the second dose of HAV vaccination

|  | **Included**  **(n=986)** | **Not included (n=140)** | ***P*** |
| --- | --- | --- | --- |
| Age, median (IQR), years | 34 (29-40) | 36 (30-42) | 0.012 |
| Male sex, n (%) | 973 (98.7) | 136 (97.1) | 0.162 |
| Men who have sex with men, n (%) | 942 (95.5) | 135 (96.4) | 0.629 |
| Weight, median (IQR), kg | 67 (60-75) | 68 (60-73) | 0.531 |
| Body-mass index, median (IQR), kg/m^2^ | 22.6 (20.7-24.7) | 22.7 (20.7-24.3) | 0.886 |
| HBsAg positivity, n (%) | 88 (8.9) | 19 (13.6) | 0.079 |
| Anti-HCV positivity, n (%) | 61 (6.2) | 10 (7.1) | 0.663 |
| Receiving immunosuppressant^a^, n (%) | 3 (0.3) | 0 (0) | 0.513 |
| Receiving cART at vaccination, n (%) | 945 (95.8) | 138 (98.6) | 0.115 |
| Lowest-ever CD4 count, median (IQR), cells/µL | 266 (133-386) | 254 (130-361) | 0.590 |
| CD4 count at vaccination, median (IQR), cells/µL | 587 (452-764) | 570 (444-745) | 0.635 |
| PVL <200 copies/ml at vaccination, median (range), log copies/mL | UD^b^ (UD-6.3) | UD (UD-4.9) | 0.453 |
| Combinations of HAV vaccine, n (%) |  |  | <0.001 |
| Havrix-Havrix | 19 (1.9) | 0 (0) |  |
| Havrix-Vaqta | 351 (35.6) | 22 (15.7) |  |
| Vaqta-Vaqta | 616 (62.5) | 118 (84.3) |  |
| Peak anti-HAV IgG titers, median (IQR), S/CO | 10.1 (7.6-11.8) | 10.6 (8.3-12.4) | 0.031 |
| Syphilis at baseline, n (%) | 262 (26.6) | 30 (21.4) | 0.194 |

^a^Including concurrent use of chemotherapy and immunomodulation agents.

^b^UD, <20 copies/mL.

Abbreviations: cART, combination antiretroviral therapy; HAV, hepatitis A virus; HBsAg, hepatitis B surface antigen; HCV, hepatitis C virus; IgG, immunoglobulin G; IQR, interquartile range; PLWH, people living with HIV; PVL, plasma HIV RNA load; UD, undetectable; S/CO, signal-to-cutoff.

**Supplementary Table S2.** Factors associated with incident seroreversion within 60 months of the second dose of HAV vaccination

|  | **Univariable** | | **Multivariable** | |
| --- | --- | --- | --- | --- |
|  | **HR (95% CI)** | ***P* value** | **AHR**^a^ **(95% CI)** | ***P* value** |
| Age, per 1-year increase | 1.04 (1.01-1.06) | 0.002 | 0.99 (0.99-1.00) | 0.974 |
| Male sex | 1.22 (0.17-8.79) | 0.840 |  |  |
| Body-mass index, per 1-kg/m^2^ increase | 1.09 (1.04-1.15) | 0.001 | 1.10 (1.04-1.16) | 0.001 |
| HBsAg positivity | 1.71 (0.95-3.08) | 0.072 | 1.53 (0.82-2.85) | 0.185 |
| Anti-HCV positivity | 1.86 (0.97-3.59) | 0.063 | 1.55 (0.70-3.41) | 0.278 |
| Receiving immunosuppressant^b^ | 4.42 (0.62-31.69) | 0.140 |  |  |
| Receiving cART at vaccination | 1.29 (0.41-4.07) | 0.665 |  |  |
| Lowest-ever CD4 count, per 10 cells/µL increase | 0.98 (0.97-1.00) | 0.004 | 0.99 (0.97-1.00) | 0.070 |
| CD4 count at vaccination, per 10 cells/µL increase | 0.99 (0.98-1.00) | 0.009 | 1.00 (0.99-1.01) | 0.956 |
| PVL <200 copies/ml at vaccination | 0.52 (0.28-0.95) | 0.034 | 0.32 (0.17-0.60) | <0.001 |
| CD4 count at the end of follow-up, per 10 cells/µL increase | 0.99 (0.98-0.99) | 0.002 | 0.99 (0.98-1.00) | 0.118 |
| PVL <200 copies/ml at the end of follow-up | 0.54 (0.17-1.69) | 0.287 |  |  |
| Combinations of HAV vaccine |  |  |  |  |
| Havrix-Havrix | Reference |  |  |  |
| Havrix-Vaqta | 1.23 (0.30-5.06) | 0.776 |  |  |
| Vaqta-Vaqta | 0.64 (0.16-2.65) | 0.539 |  |  |
| First dose of HAV vaccine, Vaqta vs. Havrix | 0.53 (0.35-0.79) | 0.002 | 0.45 (0.29-0.71) | <0.001 |
| Second dose of HAV vaccine, Vaqta vs. Havrix | 0.85 (0.21-3.45) | 0.818 |  |  |
| Syphilis during follow-up | 1.22 (0.80-1.86) | 0.349 |  |  |

^a^The HRs are the estimates of the effect of covariates on incident seroreversion after vaccination, adjusted for age, body-mass index, HBsAg positivity, anti-HCV positivity, lowest-ever CD4 count, CD4 count and undetectable PVL at vaccination, CD4 count at the end of follow-up, and first dose of HAV vaccines using logistic regression model.

^b^Including concurrent use of chemotherapy and immunomodulation agents.

Abbreviations: AHR, adjusted hazards ratio; cART, combination antiretroviral therapy; HAV, hepatitis A virus; HBsAg, hepatitis B surface antigen; HCV, hepatitis C virus; HR, hazards ratio; PVL, plasma HIV RNA load; S/CO, signal-to-cutoff.

**Supplementary Figure S1.** Evolution of serologic responses after the second dose of HAV vaccination according to the status of seroreversion at month 60 of the second dose of HAV vaccination

**Supplementary Figure S2.** Kaplan-Meier estimates of time to seroreversion after the second dose of HAV vaccination. (A) Comparison between PLWH having received different 2-dose combinations of HAV vaccines. (B) Comparison between PLWH having received different first-dose HAV vaccines.

(A)

(B)

Abbreviations: HAV, hepatitis A virus; PLWH, people living with HIV.
